# Farm-based therapeutic horticulture for people living with dementia: the Vicenza Farm Project

**DOI:** 10.3389/frdem.2026.1772835

**Published:** 2026-03-17

**Authors:** Leonardo Dalla Costa, Francesca Meneghello, Costantina Righetto, Giulio Senes

**Affiliations:** 1 Vicenza’s Alzheimer Associations: Associazione Volontariato Alzheimer Odv, Associazione Malattia Di Alzheimer E Altre Demenze A.M.A.d. Bassano del Grappa Odv, A.M.A Ovest Vicentino Odv, Associazione Veneto Malattia di Alzheimer e Demenze degenerative A.V.M.A.D., Vicenza, Italy; 2 Cure Primarie - Distretto 3, ULSS 3 Serenissima, Venezia, Italy; 3 Universita degli Studi di Bologna, Bologna, Italy; 4 Department of Agricultural and Environmental Sciences, University of Milan, Milano, Italy

**Keywords:** Alzheimer, dementia, green care farms, neuropsychology, non-pharmacological interventions, social agriculture, therapeutic horticulture, young onset dementia (YOD)

## Abstract

Dementia is a major global health challenge, with wide-ranging psychosocial and relational impacts that call for supportive, everyday interventions alongside clinical care. While advances in Alzheimer’s disease are promising, dementia includes multiple conditions, including young-onset forms, and existing pharmacological and non-pharmacological interventions still offer valuable but incomplete support, often leaving everyday psychosocial needs unmet for most people living with dementia. This Perspective describes the Vicenza Farm Project, a farm-based therapeutic horticulture program developed on social and educational farms of Northern Italy. The program offers weekly 3-h group sessions from March to October for around 8–10 participants, including people with young-onset dementia, facilitated by a multidisciplinary team of farmers, psychologists and trained volunteers. Activities follow a structured, replicable protocol that combines cognitive stimulation, seasonal gardening and farm tasks, shared breaks and closing reflection, with an emphasis on supported participation, personal agency and safe freedom. Drawing on qualitative and quantitative data from international literature on nature-based and care farming interventions, we outline how a farm-based therapeutic horticulture can help restore purpose, social connection and embodied identity while also animating rural spaces and reinforcing environmentally sensitive farming practices. We contrast the Italian policy framework of social agriculture, in which therapeutic horticulture is not yet recognized as a health intervention, with the more institutionalized Dutch model of green care farms, and we propose priorities for evaluation, integration into dementia pathways and long-term funding.

## 1 Introduction

Dementia is one of the major public health challenges of our century, affecting over 55 million people worldwide, with that number expected to more than double by 2050 (World Health Organization, 2025; Alzheimer’s Disease International, 2024). New drugs that modify the course of Alzheimer’s disease, such as monoclonal antibodies targeting amyloid, represent an important step forward, but they are not a solution for everyone: they are only indicated for selected patients in the early stages, require specialized infrastructure and monitoring, and raise substantial questions about cost and equity of access (De Strooper et al., 2025; Yakushev et al., 2025). At the same time, many communities live in environments characterized by pollution and social vulnerability, which can further increase the risk of dementia. In the Italian province of Vicenza, for example, severe PFAS contamination of water supplies has been documented, culminating in a recent criminal conviction for environmental disaster (Menegatto et al., 2025; Università degli Studi di Padova, 2025).

This Perspective presents a complementary response that is available today: farm-based therapeutic horticulture for people living with dementia. We describe the Vicenza Farm Project, a program developed on social and educational farms in Northern Italy that offers weekly 3-hour sessions of outdoor, gardening and farm-based activities to people with dementia, guided by a multidisciplinary team of farmers, psychologists and trained volunteers. We situate the project within a rural world undergoing profound transformation such as depopulation, ageing farmers, and changing in land use where social agriculture is emerging as a new way to connect care, community and sustainability (Giarè et al., 2018; Moruzzo et al., 2022; Fanelli, 2023). Based on qualitative feedback from participants and healthcare professionals, as well as international evidence on nature-based interventions, we believe that therapeutic agriculture can help people with dementia regain purpose, identity, and social connections, while revitalizing rural areas (Lu et al., 2019; Murroni et al., 2021; Whelden and Sawin, 2023; de Boer et al., 2017; Smit et al., 2022). We contrast the Italian framework of social agriculture, in which therapeutic horticulture is still not recognized as a healthcare intervention, with the more structured Dutch model of care farming for dementia, and discuss the implications for policy and practice.

## 2 Alzheimer’s disease and the limits of pharmacological and non-pharmacological treatments

Dementia, most commonly due to Alzheimer’s disease, currently affects an estimated 55–57 million people worldwide and is a leading cause of disability and dependency in older age (Nichols and Vos, 2021; World Health Organization, 2025). In 2019, the global societal cost of dementia was estimated at around US$1.3 trillion and is projected to reach US$2.8 trillion by 2030 (Alzheimer’s Disease International, 2024; World Health Organization, 2025). For families, dementia often means years of emotional strain, financial insecurity and intensive caregiving.

Over the past decades, pharmacological treatments such as cholinesterase inhibitors and memantine have provided important symptomatic benefits for many people with Alzheimer’s disease, particularly in cognition and daily functioning, even if their effects are generally modest and time-limited (Grutzendler and Morris, 2001; Reisberg et al., 2003). More recently, the development of amyloid-targeting monoclonal antibodies, such as lecanemab and donanemab, has generated enormous expectations. Phase 3 trials in early Alzheimer’s disease have shown that these drugs can modestly slow cognitive and functional decline, leading to regulatory approvals in several countries and, in 2024–2025, to the first EU authorisation of lecanemab for a restricted group of patients with early Alzheimer’s disease and confirmed amyloid pathology (Wise, 2025; De Strooper et al., 2025). These advances represent an important step forward and are rightly welcomed. Nevertheless, many individuals are diagnosed too late or not at all to benefit, and these new treatments require repeated intravenous infusions, MRI monitoring and specialised multidisciplinary infrastructure (Yakushev et al., 2025; De Strooper et al., 2025).

Alongside drugs, neuropsychological and functional interventions, including cognitive stimulation, cognitive rehabilitation, occupational therapy and multidisciplinary rehabilitation programmes, have shown beneficial effects on cognition, everyday functioning and quality of life (Abraha et al., 2017; Bahar-Fuchs et al., 2019). However, these approaches are often time-limited, require specialised staff and settings, and can be difficult to access for people living in smaller communities or with limited resources. They may also only partially address needs related to identity, meaningful occupation, contact with nature and social participation (Kim et al., 2024).

Furthermore, Alzheimer’s disease is only one of several dementias, and public as well as research attention to other forms (e.g., vascular, frontotemporal and Lewy body dementia) remains comparatively limited; moreover, even within “dementia” there is substantial heterogeneity in age, comorbidities, functional status and social circumstances. Even in an optimistic scenario of fair access to current and emerging therapies, many people will therefore continue to need additional forms of support that can be implemented flexibly in their everyday environments and communities.

## 3 Vicenza: a contaminated and ageing territory

The province of Vicenza in the Veneto region exemplifies the complex context in which dementia care unfolds. Alongside rapid industrial development, the area has been at the center of one of the largest PFAS (Per- and Polyfluoroalkyl Substances) contamination cases in Europe. High levels of PFAS have been documented in groundwater and drinking water across parts of Vicenza, Padua and Verona provinces, leading to large-scale biomonitoring programs and deep concern among residents (Menegatto et al., 2025). In June 2025, the Vicenza court handed down a historic ruling in the “Miteni” PFAS trial, convicting 11 former managers for long-term pollution of water and soil and recognizing environmental disaster and health risks (Università degli Studi di Padova, 2025).

PFAS are persistent pollutants with cardiometabolic and neurotoxic effects, and recent reviews have hypothesized that chronic exposure may contribute to increased risk of cognitive decline and dementia, although epidemiological data are still limited and sometimes conflicting (Gardener et al., 2025; Pini et al., 2023; Currie et al., 2024). This adds to a broader body of work linking air pollution and environmental exposures to neurodegenerative disease and dementia (Livingston et al., 2024; Gecegelen et al., 2025).

At the same time, Vicenza is part of a rural and peri-urban region in transformation. Across Italy, farms are fewer but larger, rural populations are ageing, and generational turnover is slow: almost 91% of Italian farms are run by farmers older than 40, and only about 9–13% of farm heads are under 40–45 years of age (Fanelli, 2023; Licciardo et al., 2023). Many smallholdings disappear, and demographic ageing and out-migration threaten the vitality of rural communities (Altamore et al., 2024).

In this environment, dementia and territorial change are intertwined: older people with cognitive impairment often remain in areas with limited services and degraded ecosystems, while families and communities struggle to find support that is both meaningful and close to home.

## 4 The Vicenza Farm Project: farm-based therapeutic horticulture for people with dementia

### 4.1 Origins and partnership structure

The Vicenza Farm Project (“Ortoterapia in Fattoria”) emerged in Northern Italy through collaboration among four Alzheimer associations, local social and educational farms, and community stakeholders. The initiative reflects wider European trends in care farming, where farms combine agricultural production with social and health services for diverse groups, including people with dementia (de Bruin et al., 2020).

Key partners in the Vicenza Farm Project include the following:

- Alzheimer associations, which provide outreach, participant recruitment, caregiver support, and psychosocial expertise, in line with evidence on the central role of such organizations in the dementia support networks (Novek and Menec, 2023; Kim et al., 2024).
- Social and educational farms, offering land, infrastructure, tools, and staff experienced in inclusive work practices.
- Health and social care professionals (e.g., psychologists and clinicians), ensuring clinical oversight and integration with existing care pathways, consistent with recommendations for multidisciplinary approaches for people with dementia (Richardson et al., 2016; Kim et al., 2024).
- Volunteers and community partners, contributing to transport, hospitality, and social integration, providing assistance and promoting a less clinical setting.

### 4.2 Participants and setting

The program is designed primarily for people living with dementia, typically under the age of 80, who have a diagnosis of dementia and sufficient mobility and health to participate in outdoor activities. In the first year of implementation, 31 participants took part across the project’s four farms (25% women), with a mean age of 69.8 years and a mean Mini-Mental State Examination (MMSE; Folstein et al., 1975) score of 17.2. Clinical diagnoses included Alzheimer’s disease (48%), vascular dementia (31%), frontotemporal dementia (9%), mixed dementia (4%), mild cognitive impairment (4%), and other diagnoses (4%). The group also includes individuals with young-onset dementia (YOD), whose age, life stage and psychosocial profile often differ from those of people with more typical late-onset dementia. Many are still of working age or have recently left employment, may have dependent children, and tend to be more physically active and sensitive to age-inappropriate environments. As a result, they may experience a sharper loss of identity and role, and a greater mismatch with traditional, building-based services designed for much older and frailer users. This inclusion of participants who are younger and more physically active than typical dementia day care users aligns with evidence that people living with YOD often seek physically demanding, socially meaningful, and non-infantilizing activities (Grunberg et al., 2022; Kim et al., 2024; Mack et al., 2025).

Sessions take place on a farm that combine agricultural production with social, educational, or therapeutic activities, a model consistent with the broader care farming literature in dementia (de Bruin et al., 2020). The participating farms are located in predominantly flat areas, which facilitates safe mobility and access for people with dementia, and they operate without the use of pesticides or other synthetic chemical products, in line with environmentally sensitive and health-promoting practices. They provide varied spaces – vegetable gardens, orchards, greenhouses, animal areas, and indoor rooms for gathering, resting, and shelter – similar to environments described in studies of therapeutic gardens and outdoor horticultural programs (Murroni et al., 2021; Borella et al., 2023). Each farm also includes a room specifically dedicated to the project, which allows activities and group meetings to continue safely in case of adverse weather. A high level of on-farm biodiversity, with different crops, plants, trees and micro-environments, enables a wide range of diverse and stimulating activities across all seasons, from sowing and planting in spring to harvesting, sensory exploration and light maintenance work throughout the year.

### 4.3 Structure and activities

As summarized in Table 1, the Vicenza Farm Project follows a structured protocol for farm-based therapeutic horticulture that can serve as a model for other agricultural settings. The protocol is designed for small groups of approximately 8–10 people living with dementia and specifies the key components of each session. Now in its second year of implementation, the project has demonstrated the feasibility of this structured approach in routine practice on social farms. From March to October, participants attend weekly three-hour sessions on the farm. Each session balances routine and flexibility:

- arrival, welcome, and informal orientation;
- cognitive stimulation activity;
- core outdoor activities (soil preparation, sowing, planting, watering, weeding, harvesting, sensory exploration);
- breaks for snacks or coffee and social interaction;
- closure and brief reflection on what has been accomplished.

**Table 1:** Protocol ad main components of the Vicenza Farm Project.

Component	Description	Illustrative examples
Target group	People living with dementia with preserved mobility and without severe behavioral and psychological symptoms.	Individuals under 80 years of age with a diagnosis of dementia, experiencing role loss, social withdrawal, or apathy. 8–10 participants.
Setting and infrastructure	Farms integrating agricultural production with social and educational activities.	Flat land, vegetable gardens, orchards, greenhouses, accessible bathroom, indoor common rooms in case of bad weather.
Frequency and duration	Weekly farm-based sessions during the temperate months of the year.	Three-hour group sessions (from 9:00 a.m. to 12:00 p.m.) from March to October with a summer break.
Main activities	Nature-oriented and farm-based tasks tailored to abilities and seasons. Nature walks and sensory stimulation. Group cognitive activities.	Follow the entire vegetable production cycle: sowing in seedbeds, soil preparation, planting, irrigation, weeding, harvesting vegetables, processing.
Facilitation team	Multidisciplinary group combining clinical, agricultural, and community competences.	Activities conducted by a neuropsychologist, a farm worker, and trained volunteers. Monthly organizational meetings.
Session structure	Balance of predictable routine and flexible adaptation to participants’ needs and weather.	Arrival and welcome, diary, sensory walk, outdoor work, snack or coffee break, closing reflection on the activities accomplished.
Community dimension	Social events to reduce stigma and isolation associated with dementia.	Farm parties with mutual aid groups for caregivers and recreational activities for participants. Final buffet inviting local authorities.
Underpinning principles	Person-centered care and community-embedded care model.	Emphasis on autonomy, usefulness, identity, and inclusion rather than on deficits or “patient” roles.

In Table 2, we illustrate how these components are organized within a typical morning session, showing the sequencing and timing of activities across the three-hour program. Activities are adapted to the season, weather, and participants’ abilities, with emphasis on participation rather than performance. This structure is broadly consistent with intervention designs described in controlled studies of horticultural activities in dementia, which combine regular, seasonal outdoor sessions with simple, participatory tasks (Borella et al., 2023; Wang et al., 2024).

**Table 2:** Protocol ad main components of the Vicenza Farm Project.

Phase	Main objectives	Examples (March 19, 2025)
Welcome	Ensure safety, orientation and a feeling of being valued and expected.	Participants place their backpacks in the same place and retrieve the tag with their name on it.
Diary and reality orientation	Support orientation and spontaneous memory of previous sessions.	We begin with a temporal orientation (day, month, year, season, weather) and review the activity carried out in the last lesson, “summer vegetable seedbed.”
Cognitive stimulation based on the activity of the day	Stimulate language, creativity, reasoning and autobiographical memory; build cohesion.	To stimulate language, participants are asked to choose between “rose” and “sunflower” and explain their preference. At the end, a survey is conducted to choose the group’s favorite flower and create cohesion.
Walk around the farm	Encourage safe movement, warming up for the activity, sensory stimulation and contact with nature.	Initial walk around the farm, pointing out the new flowers that have bloomed and reminding participants of the names of the fruit trees.
Main activity	Promote meaningful, hands-on participation and sense of usefulness.	“Sowing flowers in seedbeds”: participants are paired with volunteers. The activity is broken down into several steps: mixing the soil, filling the seed trays with soil, making holes with the finger, inserting the seeds, watering, and labeling with the name and date.
Break	Offer rest, pleasure and informal social interaction.	Herbal tea and some cookies while sitting at the table in the garden.
Group recap	Consolidate the experience and support reflection on the day.	Together, we write the daily journal and how the activity went.
Closing conversation	Gently transition toward going home, containing fatigue and restlessness.	Final activity, the first memory associated with flowers: for example, memories of their childhood hometown.

### 4.4 Multidisciplinary facilitation

Sessions are guided by a multidisciplinary team comprising at least one psychologist, one or more farmers, and trained volunteers. This mix of clinical and non-clinical expertise resonates with recommendations for psychosocial interventions in dementia, which emphasize interdisciplinary collaboration and attention to both clinical and social dimensions (Richardson et al., 2016; Tong et al., 2025; Kim et al., 2024). Farmers bring practical knowledge of land, animals, and agricultural rhythms, as well as a “working” culture that normalizes participation and frames activities as real, useful work rather than therapy. They are also responsible for day-to-day risk management on the farm, identifying and mitigating potential hazards (e.g., tools, uneven ground, animals) while preserving as much autonomy as possible for participants.

The psychologist plays a specific role in supporting the person-plant relationship, using horticultural tasks not only as occupation but as a medium for emotional expression, reminiscence, sensory stimulation and identity work. Studies on therapeutic horticulture and therapeutic gardens highlight that the intentional facilitation of people-plant interactions such as inviting participants to care for a specific plant, observe growth over time, or use plants to trigger memories can enhance engagement, reduce stress, agitation, fatigue, and support wellbeing in people with dementia (Righetto, 2015; Koura and Ikeda, 2016; Lu et al., 2019; Borella et al., 2023; Wichrowski and Moscovici, 2024). Although internationally there is increasing discussion of “horticultural therapists” as specialized professionals, in Italy this profile is not yet formally recognized in health or professional regulations; in practice, psychologists and other clinicians integrate horticultural methods into their existing roles within social agriculture projects.

Volunteers receive annual training in therapeutic horticulture and dementia care and play a predominantly practical, relational role on the farm. In many activities, volunteers work one-to-one alongside participants, offering step-by-step support, encouragement, and gentle prompts, but without replacing them or taking over the task. This kind of “supported participation” is consistent with evidence that relational scaffolding, rather than substitution, helps people with dementia remain engaged and feel competent in everyday activities (Phinney et al., 2007; McCabe et al., 2018). Volunteers help maintain a cheerful, welcoming atmosphere in the group, which is particularly important in an open, outdoor context where attention and energy naturally fluctuate (de Boer et al., 2017; Rosteius et al., 2022). Their presence also increases supervision and safety in the farm environment, allowing participants to move more freely while still being closely accompanied, a feature highlighted as crucial in studies of green care farms and social farming for vulnerable groups (Hassink et al., 2010; Moruzzo et al., 2022). This is particularly relevant because some people living with dementia may move around the environment in response to unmet needs, reduced navigational abilities, or environmental features, especially in unfamiliar outdoor settings, making supportive accompaniment important for safety while preserving autonomy (Graham, 2017).

## 5 Policy and international context: social agriculture and care farming

The Vicenza Farm Project develops at the intersection of social agriculture and dementia care, two worlds that still rarely meet in formal policy.

In Italy, animal-assisted interventions (“pet therapy”) have been progressively standardized through national guidelines and State-Regions agreements, with clear training requirements and quality standards (Ministero della Salute, 2015). This has facilitated their recognition within regional health services. By contrast, therapeutic horticulture is not yet formally recognized as specific therapeutic intervention in national health policy: there are no official clinical guidelines or certified training pathways, and projects are usually funded through local or regional initiatives, social-agriculture measures or time-limited grants, rather than stable health budgets. They occupy a hybrid space between health, social care and rural development.

At the same time, social agriculture is clearly defined in Law 141/2015 and promoted in regional rural development programs. Social farms are recognized as actors that can provide social inclusion, educational activities and socio-health services using agricultural and rural resources (Giarè et al., 2018; Moruzzo et al., 2022; Ministry of Agriculture, Food and Forestry Policies, 2015). This gives projects like the Vicenza Farm Project a legal and cultural home, but not yet a secure place in dementia care pathways and funding schemes.

In the Netherlands, by contrast, green care farms have become structurally embedded in long-term care. More than 1,000 green care farms exist, with a substantial proportion offering day or residential services for people with dementia and, in some cases, 24-h nursing home care funded through long-term care and social support schemes (de Boer et al., 2017; Smit et al., 2022; Rosteius et al., 2022). Studies show that residents on green care farms are more physically active, spend more time outdoors and engage in more social interaction than those in conventional nursing homes, with family caregivers reporting high satisfaction and more homelike environments (de Boer et al., 2017; Smit et al., 2022).

We see the Vicenza Farm Project as part of this broader “green care” movement, but developing along a different institutional trajectory. While Dutch care farming is funded through long-term care and social support schemes, Italian farm-based dementia projects rely on a patchwork of rural, social and project-based funding. For therapeutic horticulture to move from promising practice to stable service, Italy will need to bridge the gap between social-agriculture policy and dementia-care planning, explicitly recognizing nature-based interventions as components of comprehensive dementia strategies.

## 6 Conclusion

In a world where new Alzheimer’s drugs offer hope but also raise questions of cost, equity and feasibility, farm-based therapeutic horticulture offers something different: a low-tech, community-embedded response that is available now. The experience of the Vicenza Farm Project suggests that farms can become places where people with dementia are not only cared for, but also active participants in meaningful, shared work with nature. At the same time, this model is relatively low-cost, relies heavily on organised volunteering alongside farmers and professionals, and is built on a structured but simple protocol, making it a realistic and replicable option for other agricultural and community contexts that wish to develop similar programmes.

This Perspective argues for complementarity across levels of care. Pharmacological approaches may benefit some people by targeting biological pathways, while interventions such as therapeutic horticulture can foster participation, purpose and social connection across diagnoses and stages. At the same time, the project reminds us that dementia is deeply territorial, unfolding in specific places – such as an industrialized, PFAS-contaminated and rapidly changing province like Vicenza – where environmental exposures, rural transformation and service availability intersect. Social agriculture and care farming suggest that rural worlds in upheaval are not only sites of loss, but also laboratories for new forms of care that combine health, inclusion and environmental sustainability.

Looking ahead, we see three priorities:

1. Evaluation – More systematic research on farm-based therapeutic horticulture for dementia, including mixed-methods designs, longer-term follow-up, economic analysis, and reliable, standardized data collection across projects and sites.
2. Integration – Clearer pathways linking memory clinics, primary care and Alzheimer associations to social and educational farms, with criteria for referral, feedback and collaboration.
3. Recognition and funding – Policy frameworks that build on Law 141/2015 and rural development programs to explicitly include therapeutic horticulture and care farming in dementia care strategies and financing.

Projects like the Vicenza Farm Project show how care can still be grown: in the soil, in relationships, and through the shared work of making rural spaces places of life – not only of memory – for people living with dementia.

## Data Availability

The original contributions presented in the study are included in the article/supplementary material, further inquiries can be directed to the corresponding author.
